# Family Order

**Published:** 1858-05

**Authors:** 


### HALL'S JOURNAL OF HEALTH.

OUR LEGITIMATE SCOPE IS ALMOST BOUNDLESS: FOR WHATEVER BEGETS PLEASURABLE AND HARMLESS FEELINGS, PROMOTES HEALTH; AND WHATEVER INDUCES DISAGREEABLE SENSATIONS, ENGENDERS DISEASE.

*We aim to show how Disease may be avoided, and that it is best, when sickness comes, to take no Medicine without consulting an educated Physician.*

VOL. VI.] MAY, 1858. [No. 5.

### FAMILY ORDER.

What a delightful thing it is to know, that from cellar to garret, there is not a hiding-place for the smallest piece of dust, or dirt, or rubbish; that everything about you is in a cleanly condition; that every piece of clothing is in its usual and proper place; that of the multitudinous articles of domestic convenience and necessity, there is not one which is unfit for immediate use, not one that could not have hands laid on it at any hour of the day or night; to have children and domestics so well drilled, that none of them will fail, in a month, to put a thing in its proper place, the moment it ceases to be used; to have all know that doors are to be closed behind them; that the feet are to be well wiped on the door-mat; that nothing is to be stepped over; that any unsightly thing is to be removed by the first discoverer, by whatever accident placed there; that every garment be left at night at the same spot, and arranged in such a way, that the first one touched is the first to be put on; that no one is to be called twice to either meal or to get up in the morning; that each one study to spare labor to another, soiling as few garments as is compatible with faultless cleanliness; to be willing to incommode one's self, rather than impose unnecessary labor on cook, laundress, nurse, seamstress, or housemaid; that children be self-denying as to one another, loving as to parents, deferential as to guests, and courteous towards servants! To have these things requires a wife, who is orderly, systematic, and industrious. It requires a great deal of patience to develop tnem in children and hirelings. Yet it can be done, for there is a house of that sort opposite our office window, and there is another of the same kind opposite that house. Notable wives are they, worthy of a long hunt, and rich without a dollar. It is true, that in cases of the kind which have come under our notice, there is rather a preponderance of capsicum, a mite more than is compatible with perfect felicity; but even gold has some alloy.

What a terrible life must that unfortunate husband lead, whose "partner" is so affectionate and amiable, that she cannot bear to be cross, or to reprove anybody about her; who is willing to let all have their own way; who never can see the use of having everything "just so;" who can never tell where anything is; whose practice is to fit for use when wanted, on the ground that if it is never wanted, there is so much labor saved; who never lays a garment away smoothly, because if it has forty thousand wrinkles in it, it can't be stiff, and is therefore more pliable; who believes that much exercise is dangerous, and prevents that rotundity and fullness of muscle, of cheek and limb, which all admire; who is satisfied in her own mind, that if everything is allowed to take care of itself, there will be no need of her taking care of it, unnecessary labor being a clear loss; who does not believe in "shams," therefore will not have the ceilings whitewashed every spring, because whitewash only covers up the blackness under it; who says it is wasteful to sweep a carpet, because it wears it out, and if let alone, the dirt will hide itself "*underneath;*" who has always been under the impression that dampness in a dwelling is unhealthful, and therefore never has the stairs or floors scrubbed; who avers the uselessness of having the painted wood-work washed, as it takes off the paint, and if it was not intended that the paint should remain there, it should not have been put on; who never sews on a button for her husband, because, if he has to sew them on himself, he will be more careful not to twist them off; who does not employ a seamstress, because it is expensive, then sits and sews from morning till night, until she is laid up for want of exercise, then must have an extra servant for a nurse, which, with the doctor's bill, would pay a seamstress for a year's work; who sews until midnight, because in the morning she always feels sleepy, and it takes until breakfast time to get her sleep out; who spends half her time in showing the housemaid how to do things, Biddy looking on with great soberness (as she used to do in her "*last place*," and will do again), because it is a fine thing for the mistress to earn the wages of the maid; who don't like to go down into the kitchen to "look after things," because it looks close and mean to the servants; who hates to lock up things, because it is unfeeling to let the "help" see that you are suspicious, when you have no evidence that they are dishonest; that it is no use to be so saving of food and fuel, for then, scavengers and beggars would have no encouragement to go around and get an honest living; who will at times exert themselves beyond their ability, because there is work to be done, and they can't help it, if they are made sick by it, somebody must be sick, or the doctors would starve; who will tease their husbands for this that or the other coveted item, because such and such a one has just bought one. "But they live on their income, and we are in moderate circumstances." "I don't believe in denying ourselves for the sake of our children, let them tug and toil for themselves." Such is the line of argument in many households, the result, in too many cases, being the destruction of family peace, comfort, and enjoyment; it is thus that many an ambitious, economical and industrious young husband has been discouraged into idle habits, or driven to spend his "evenings out" in societies, clubs, bar-rooms, and brothels, to end in a drunkard's death, a family unprovided for, in a long widowhood of toil, and penury, and want, bringing to mother and children that crushing out of all life's hopes which is the certain precursor of wasting disease and premature death.

Let mothers, therefore, as the best means of saving their daughters from wreck and ruin, make it their daily care to bring them up in such a manner, that when they enter practical life, they may be able to perform well the responsible duties of wife, mother, matron. Such a mother honors herself, lays a broad foundation for the happiness of her children, and her children's children, and is one of society's best benefactors.

In view of these facts, we earnestly advise young men to let the character of the mother have a large influence in determining their choice of a wife, a choice which makes or mars the lot of life, and often moulds the destiny beyond. With a good wife, a man may be comparatively happy under all circumstances; without one, he cannot be happy in any.

